# Updated review of research on the role of the gut microbiota and microbiota-derived metabolites in acute pancreatitis progression and inflammation-targeted therapy

**DOI:** 10.7150/ijbs.108858

**Published:** 2025-01-20

**Authors:** Qiang Liu, Kaiyi Ruan, Zihui An, Lingyun Li, Cong Ding, Dongchao Xu, Jianfeng Yang, Xiaofeng Zhang

**Affiliations:** 1 Department of Gastroenterology, Affiliated Hangzhou First People's Hospital, Westlake University School of Medicine, Hangzhou 310058, China.; 2 Key Laboratory of Integrated Traditional Chinese and Western Medicine for Biliary and Pancreatic Diseases of Zhejiang Province, Hangzhou 310006, China.; 3 Hangzhou Institute of Digestive Diseases, Hangzhou, Hangzhou 310006, China.; 4 Zhejiang University School of Medicine, Hangzhou 310058, China.

**Keywords:** intestinal microbiota dysbiosis, intestinal barrier integrity, immunomodulation, crosstalk, therapeutics, immune homeostasis

## Abstract

Acute pancreatitis (AP) is characterized by autodigestion of the pancreas, and some patients may rapidly progress to systemic inflammation, pancreatic necrosis, and multi-organ failure. Numerous studies have detailed the bidirectional communication networks between the pancreas and the intestinal microbiota, as well as its metabolites. Such crosstalk affects the progression of AP and recovery through intestinal barrier disruption. Furthermore, advances in experimental research and clinical studies have indicated that gut microorganisms exhibit distinct alterations in response to different levels of severity and etiologies of AP. This information has greatly expanded our knowledge of the role of the gut microflora and microbial metabolites in the pathology of disease and has reinforced the basis of therapeutic approaches that target candidate intestinal microbiota. In this review, we aim to provide an overview of the composition and diversity of the gut microbial community, to highlight the candidate bacteria and microbiota-derived metabolites responsible for AP, and to elucidate their interactions with and regulation of immune-relevant receptors in intestinal epithelial cells (IECs) in the host. Future research should focus on identifying and characterizing AP-associated bacterial strains, elucidating their distinct pathogenic mechanisms across different etiologies and stages of AP, and leveraging these insights to develop preventive and therapeutic strategies.

## 1. Introduction

Acute pancreatitis (AP) is a serious inflammatory disease with multiple complications and a high fatality rate, with an annual incidence of 34 cases per 100,000 people worldwide 1. Nearly 80% of patients are diagnosed with mild AP (MAP), which is self-limiting for two weeks with minimal supportive caret; however, patients with severe AP (SAP) experience rapid aggravation, with a high mortality rate of nearly 30% 2. However, the pathophysiological mechanisms underlying SAP remain elusive, and precise pharmacological therapeutic strategies with reliable and stable efficacy for the treatment of SAP are lacking. Currently, accumulating evidence suggests that the diversity and composition of the gut microbial ecosystem are strongly associated with the initiation, progression and treatment response of SAP 3, 4. Numerous studies with diverse approaches have investigated reversing microbiota dysbiosis; these strategies have favorable therapeutic outcomes in AP patients 5, 6.

The early stage of SAP induces gut microbiota dysbiosis and destruction of the intestinal barrier, which in turn exacerbates the severity of AP in the advanced stage 7-9. Gut microorganisms are involved in various immunopathological mechanisms and impact gut immune homeostasis by regulating the production of metabolites and interacting closely with immune-related receptors and transporters, which are also present in the gut 10. However, the impact of gut bacterial dysbiosis and dysfunction on SAP, as well as the role of specific intestinal bacterial species and their metabolites in the etiology of SAP, remains largely unclear. In this review, we systematically describe the distinct spectrum and alterations of SAP-related intestinal microbiota (excluding viruses, bacteriophages, fungi and parasites) and microbiota-derived metabolites to explore potential therapeutic strategies related to reversing the intestinal microbiota dysbiosis.

## 2. Roles of the intestinal microbiome and metabolome in the pathogenesis, disease progression, and recovery of AP

Because of the connections among the pancreas, the duodenal papilla via the pancreatic duct, intestinal dysmotility, ischemia‒reperfusion and oxidative stress during the aggravation of AP, enterobacterial dysbiosis and translocation are thought to promote secondary infection and sepsis in AP patients and increase the risk of multiple organ failure (MOF), mortality, and the length of hospital stay 6. Further studies on the role of microorganisms in AP progression have shown that infected pancreatic necrosis originates from the translocation of commensal gut bacteria into the pancreas and the pathogen-associated molecular patterns (PAMPs) of AP-associated tissue injury via the binding of intestinal bacteria with pattern recognition receptors, which aggravate symptoms 11, 12. These studies indicate that gut bacteria play important roles in the progression of SAP; thus, revealing the role and mechanism of microbial targets and their metabolites in different stages and etiologies of SAP is necessary.

### 2.1 Bacterial taxa associated with AP in animal studies

Various studies with diverse approaches, such as those that utilize antibiotic intervention 13, germ-free mice 14, fecal microbiota transplantation (FMT) 15, and the genetic modification of mice 16, have been conducted to investigate the effectiveness of targeting the gut microflora in the treatment of inflammatory diseases, including AP. Although many microbial alterations have been identified by comparing microbiomes between patients with AP progression and normal controls, there is no consensus on the causal relationship between the gut microbiota and AP. To eliminate concerns about potential false-positive results and gain a comprehensive spectrum of the intestinal microbiome in AP, we focused primarily on the microbiota that underwent significant changes and consistently impacted AP in at least two independent metagenomic datasets from animal and clinical studies. Through this research, links between fourteen commensal bacteria and the progression of AP in animal models were established (Table 1).

Among the fourteen AP-relevant flora, the abundances of Escherichia-Shigella, Enterococcus, Proteobacteria, and Firmicutes were increased in AP patients, and the Prevotella, Bacteroides, Bifidobacterium, Lachnospiraceae, Muribaculaceae, Parabacteroides, Helicobacter, Lactobacillus, Akkermansia, and Actinobacillus were decreased, reflecting their consistent effectiveness in mediating AP progression 16-22. Escherichia-Shigella and Enterococcus are the most widely reported opportunistic pathogens associated with inflammation and intestinal barrier disruption in AP 23, 24. Several studies reported that the abundance of Escherichia-Shigella in AP patients was approximately 50- and 100-fold greater than that in healthy controls, which was strongly correlated with local or systematic inflammation, the invasion of intestinal epithelial cells (IECs) and the exacerbation of AP 4, 15. Interestingly, dysbiosis of the gut microbiota, triggered by Escherichia-Shigella or Enterococcus, stimulates massive differentiation of Tregs, causing to a Treg/Th17 imbalance and inhibiting Teffs and IELs, which provides an opportunity for secondary pancreatic necrotic tissue infections 12, 25. The antioxidant activity of chitosan oligosaccharides can significantly reduce the number of Escherichia-Shigella and Enterococcus bacteria to alleviate the progression of AP 26. An increased abundance of Escherichia-Shigella in acute necrotizing pancreatitis (ANP) patients augments the endoplasmic reticulum stress response and disrupts intestinal tight junctions (TJs) through activation of the TLR4/MyD88/p38/NF-κB signaling pathway 27. In addition, high levels of Escherichia-Shigella in the gut increase the serum levels of proinflammatory cytokines and aggravate intestinal permeability. Interestingly, Enterococcus was once considered to have low virulence, while it was strongly associated with new/persistent organ failure and increased mortality in ANP 28. Moreover, vancomycin-resistant Enterococcus strains isolated from the feces of SAP patients presented a high degree of homology with peripancreatic effusion samples, indicating the occurrence of parenteral transmission 29, 30. A recent study revealed that Enterococcus secretes protease gelatinase, which mediates virulence through regulating host tissue degradation and the immune response 31. Therefore, we hypothesized that the transfer of Enterococcus from the intestinal tract to damaged tissues and systemic circulation is a causal factor in the aggravation of AP.

Enteral nutrition and probiotics are thought to significantly modulate inflammation in both mice and rats, indicating that the gut microbiota serves as a mediator of these therapeutic effects. Tang *et al.* reported that colonization with *Bifidobacteria*, particularly *B. animalis,* and the administration of its metabolite lactate can alleviate inflammation and improve the survival rate, thereby highlighting the potential therapeutic value of modulating the commensal gut flora and microbiota-derived metabolites 20. Additionally, Li *et al.* reported that a decreased abundance of endogenous *Bacteroides* in the gut microbiota impaired taurine production and increased inflammatory factor release in the colon, which triggered neutrophil extracellular trap formation 13. Furthermore, the abundance of the core gut microbiota *Lachnospiraceae* 32, which are the main producers of short-chain fatty acids (SCFAs), was significantly decreased in patients with ANP and SAP 22. Qingyi decoction, a commonly utilized traditional Chinese medicine for the treatment of SAP, can alleviate inflammation by increasing the abundance of SCFA-producing bacteria, such as *Bacteroides* 14*.* However, the changes in the abundances of several bacteria are still controversial. For example, the relative abundance of *Lactobacillus*, which is extensively used in a variety of commercial products 33, is strongly associated with the amelioration of pancreatic and intestinal damage 16, 19. Beneficial bacteria (e.g., *Lactobacillus reuteri* and *Bifidobacterium*) contribute to the proliferation and renewal of intestinal stem cells and the restoration of TJs by activating the Wnt/β-catenin pathway; this activation eventually attenuates SAP-mediated disruption of intestinal homeostasis and the pancreatic inflammatory response 34-36. Paradoxically, two independent studies indicated that the abundance of *Lactobacillus* was increased in duodenal mucosal biopsy samples from C57BL/6 mice 17 and feces from AP rats 5. Hence, to elucidate the association between different severity or etiology of AP and alterations in intestinal microflora, it is imperative to conduct further studies using animal models, which will enable a detailed exploration of the dynamic changes in microflora throughout the progression of AP.

### 2.2 Bacterial taxa associated with AP in human studies

Compared with those in healthy donors, significant structural shifts in the gut microbiota of AP patients have also been observed, as well. Among the variations in microorganisms associated with AP, ten gut microflora with consistent effects on AP were identified, and for additional information on the effects of dysbiosis of candidate bacteria in AP patients, please refer to Table 2. Interestingly, six candidate bacteria for AP, namely *Escherichia-Shigella, Proteobacteria, Enterococcus, Bacteroides, Bifidobacterium,* and *Prevotella*, have been found to be closely related to disease progression in both AP patients and experimental models.

In addition to the common features of intestinal microbial dysbiosis in AP, patients have distinct views of intestinal metagenomics that are related to the different levels of severity and pathogenic factors of AP 37. Alterations in the gut microbiota may contribute to the severity of AP. For example, patients with SAP presented higher levels of AP-enriched species than did patients with MAP or MSAP, and the degree of microbiota alterations revealed distinctive patterns among different etiologies 38. Tan *et al.* categorized AP patients into two groups on the basis of the similarity of their gut microorganisms to those of healthy donors, and the dissimilar group had higher incidences of MOF, infection-related complications, and APACHE scores, which indicated that altered fecal microbial composition in patients is a pivotal mechanism that contributes to the progression from MAP to SAP 39. In addition, SAP patients, but not MAP patients, presented increased abundances of *Acinetobacter* and *Stenotrophomonas* and decreased abundances of *Acteroides, Bifidobacterium* and *Gemella* 4, 18*.* Furthermore, alcoholic AP increased the abundance of *Actinobacteria* and decreased the abundance of *Bacteroidetes* 40. Additionally, compared with biliary AP patients, HTGP patients presented greater abundances of *Escherichia-Shigella* and *Enterococcus*, and the variation in these bacteria was correlated with the severity of disease and patient prognosis 41. These findings indicate a strong association between disrupted gut microbiota and the varying severity and etiological factors of AP, although the causal relationship still warrants further investigation.

### 2.3 Animal and human metabolomics studies revealed that the inflammatory response to AP is precisely orchestrated by microbiota-derived metabolites

Alterations in the levels of intestinal microorganisms, as primary determinants of metabolite production, are typically accompanied by changes in metabolomic profiles, contributing to the modulation of the physiological effects on the host organism 42. As shown in Table 3, we reviewed the differentially abundant metabolites in serum 43, pancreas 44 and fecal 45 metabolomics matrices from murine AP models; these results may elucidate the relationships between metabolites and AP.

SCFAs produced by anaerobic bacteria, such as *Bifidobacterium* and *Lactobacillus*, act as "peacekeepers" in AP by maintaining TJs proteins in IECs, suppressing the production of proinflammatory factors 46, regulating commensal resistance ^3^, and restoring intestinal barrier function 47. Lactulose effectively alleviated the inflammatory response in MSAP patients by restoring intestinal function and increasing the production of SCFAs 48. In agreement with the clinical metabolome data, both oral and systemic butyrate supplementation decreased bacterial dissemination and reversed the microbiota alterations in the ANP model 15. Butyrate, a core metabolite produced by *Clostridium*, drives the differentiation of monocytes into macrophages by inhibiting histone deacetylase 3 activity to augment the antimicrobial barrier in the host 49. SCFAs inhibited the expression of HDAC and NF-κB in innate immune cells and activated PPAR-γ, leading to increased expression of ILC3s and the secretion of anti-inflammatory factors such as IL-10, IL-22, and TGF-β. SCFAs influence T and B cells through interactions with GRP41, GRP43, and GRP109A; stimulate the expression of mTOR, Foxp3, and Ahr; reduce the numbers of Th1 and Th17 cells; and thereby preserving homeostasis and mitigating inflammation 50, 51. Additionally, SCFAs trigger the generation of intestinal TJs, such as ZO-1 and claudin-3, by activating the PI3K/AKT/mTOR pathway in IECs, which results in increased secretion of RegIIIγ and β-defensins to improve the intestinal barrier 52, 53. Although increasing evidence has demonstrated that SCFAs exert beneficial effects on multiple diseases, including ameliorating AP, a recent study revealed that all abundant species in SAP belong to SCFA-producing taxonomic families, which might provide an explanation for the failure of SCFA-containing probiotics in SAP 54. Thus, further studies are needed to fully understand the dynamics and functions of SCFA-producing species, perform targeted metabolomics, and explore the effects of probiotic interventions in AP 55.

In addition, tryptophan metabolites, which are specific ligands that bind to the aryl hydrocarbon receptor (AhR), stimulate the expression of IL-22, which further induces AhR activation and the formation of a positive feedback loop to ameliorate pancreatic injury and promote IEC homeostasis 56. *Lactobacillus* enhances tryptophan metabolism by increasing the production of AhR ligands, resulting in the promotion of intestinal barrier functions and increased secretion of incretin hormone 57. Notably, there is crosstalk between arginine metabolism and tryptophan metabolism. *Lactobacillus* promotes the synthesis of L-ornithine by promoting arginine metabolism, and this metabolite facilitates the activation of tryptophan metabolism in IECs, leads to the production of the AhR ligand L-kynurenine, and induces RORγt^+^IL-22^+^ILC3 cell differentiation 58. Moreover, supplementation with tryptophan metabolites to restore the level of tryptophan metabolism in AP reduces the severity of AP via anti-inflammatory effects and a reduction in macrophage and neutrophil infiltration 19, 59.

Numerous studies have shown that intestinal bacterial and microbiota-derived metabolites are involved in functional and intestinal barrier integrity, but their exact mechanism in AP is still unclear. Hence, given the distinctive nature of intestinal bacterial and microbiota-derived metabolites and their unique functional roles, delving deeper into their potential mechanisms and contributions is imperative.

## 3. The severity of AP is closely associated with intestinal barrier injury

### 3.1 Physical intestinal barrier integrity

**Figure 1:**
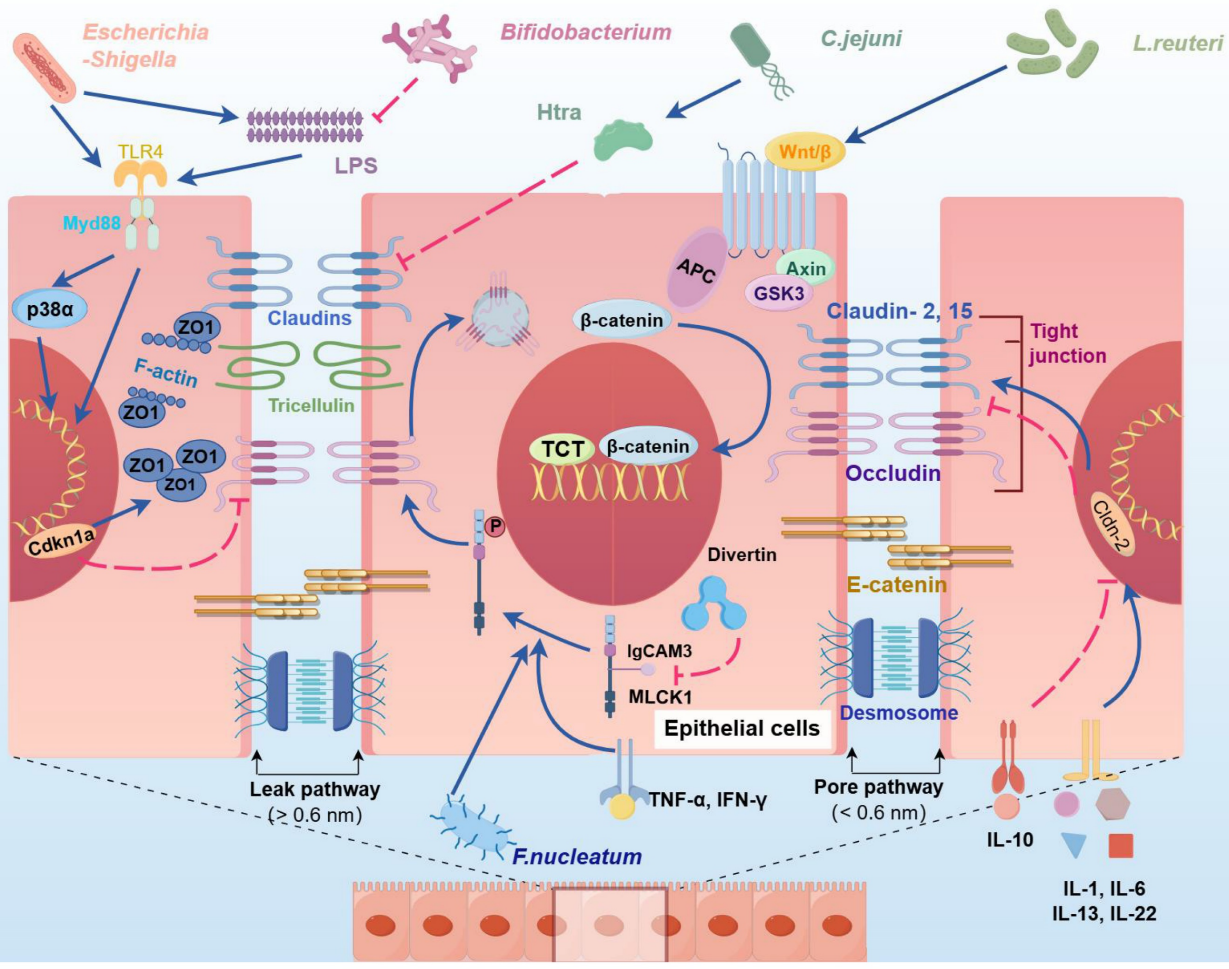
**Effects of intestinal bacteria and their metabolites on physical barriers.** The intercellular gaps between IECs are blocked by TJs, AJs and desmosomes. TJs are primarily composed of ZO-1, claudins, and occludins. Pathogenic bacteria and their cytotoxic byproduct, LPS, suppress occludin expression and induce ZO-1 aggregation by activating the TLR4/MyD88/p38 signaling pathway, upregulating Cdkn1a, and triggering NF‒κB signaling, ultimately disrupting intercellular junctions. *C. jejuni*-secreted HtrA compromises the epithelial barrier by inhibiting Cldn8 expression, thereby increasing paracellular permeability. In addition, IL-1, IL-6, and IL-13 further increase paracellular permeability by promoting the expression of Cldn2. The invasion of *Fusobacterium nucleatum* and the secretion of TNF-α, IL-1β, and IFN-γ activate MLCK1, leading to occludin internalization via myosin light chain phosphorylation, which increases intestinal permeability and facilitates gut microbiota translocation. Divertin, a small molecule, can specifically bind to the IgCAM domain in MLCK1 to inhibit its function. Conversely, probiotics such as* L. reuteri* and *Bifidobacterium* activate the Wnt/β-catenin pathway, promoting the proliferation of intestinal stem cells and restoring intestinal TJs. (Cdkn1a: cyclin-dependent kinase inhibitor; IECs: intestinal epithelial cells; TJs: tight junction; AJs: adherens junction; LPS: lipopolysaccharide; ZO-1: ZO1 tight junction protein; MLCK1: myosin light chain kinase).

The physical intestinal barrier is composed of a layer of IECs, including enterocytes, enteroendocrine cells (ECs), goblet cells (GCs), and Paneth cells (PCs), which play essential roles in mediating intestinal permeability; maintaining the equilibrium of the intestinal barrier; and impeding invasion by bacteria, viruses, and endotoxins 60. The permeability of the pore pathway is regulated by claudin, which modulates the formation of channels or barriers to respond to distinct gut conditions at TJs (Figure 1).

The physical barrier of the intestine is disrupted during AP aggravation, resulting in increased intestinal permeability and contributing to bacterial and toxin invasion and the translocation of bacteria from the bowel lumen to the portal circulation and mesenteric lymphatics 12, 17, 48. The extensive proliferation of pathogenic bacteria suppresses the expression of TJ-related proteins through PAMPs, thereby mediating the translocation of harmful bacteria and exacerbating inflammatory responses 61. For example, *Campylobacter jejuni* and *Helicobacter pylori* secrete a series of virulence factors, such as high-temperature requirement A (HtrA), to specifically inhibit claudin-8 in TJs; purified HtrA can cleave claudin-8 and occludin in vitro, thus promoting the paracellular translocation of the microorganism 62, 63. Moreover, several bacteria secrete lipopolysaccharide (LPS) to inhibit the expression of ZO-1 and occludin in IECs, which can induce the aggregation of ZO-1 at cell junctions and alter its spatial distribution, resulting in a substantial increase in paracellular permeability and epithelial barrier damage 64.

### 3.2 Chemical intestinal barrier integrity

The chemical barrier of the intestine is primarily composed of the mucus layer on the surface of IECs, which serves as the first line of defense against pathogens. This barrier includes digestive fluids and mucus secreted by GCs, as well as antimicrobial peptides (AMPs) produced by PCs. The intestinal microbial composition and structure are closely related to the formation of intestinal mucus, and they interactively and cooperatively impact the physiology of the host. Excessive inflammatory responses damage the mucus layer, leading to a reduction in AMPs and an increased likelihood of pathogen penetration through the mucus barrier, resulting in secondary infections and complications in patients with AP.

On one hand, GCs serve as the primary cellular source within the mucus layer, which constitutes the initial barrier of the intestine and is also involved in modulating the intestinal immune response. A recent study revealed that, compared with ANP mice, MUC2-deficient mice experienced more severe pancreatic injury, inhibited colonic antimicrobial peptide β-defensin-2 expression (βD2), and increased bacterial translocation, with MUC2 supplementation partially reversing the damage and emphasizing the importance of intestinal mucus integrity for pancreatic function 65. Under inflammatory conditions, MUC2 maintains intestinal homeostasis by promoting the expression of the antimicrobial peptide βD2, which effectively affects gram-negative bacteria and Candida 66. Commensal intestinal bacteria and their metabolites can directly or indirectly regulate GCs through various mechanisms, contributing to the maintenance and regulation of the intestinal mucosal barrier. *Lactobacillus rhamnosus* induces the expression of 5-HT4R and MUC2 to promote the functions and differentiation of GCs and modulate the microbial community in the gut 67. SCFA-producing bacteria, such as *Akkermansia muciniphila*, *Bacteroides*, and* Parabacteroides*, generate a diverse range of sulfatases, which allow them to interact with host mucins via the metabolism of the O-glycan domain of MUC2 as an endogenous energy source 68. However, findings concerning the relationship between *Akkermansia muciniphila* and AP are conflicting. In SAP models, a significant increase in *Akkermansia muciniphila* was observed, with a 20-fold increase in the cecal mucosa and a 100-fold increase in the colonic mucosa; however, its abundance decreased following sodium butyrate supplementation 69, 70. In contrast, several studies have reported that the abundance of *Akkermansia muciniphila* significantly increases after treatment for SAP, alleviating inflammation by specifically secreting the membrane protein Amuc_1100, which regulates host tryptophan metabolism and the composition of the gut microbiota 65, 71. The dual role of *Akkermansia muciniphila* in AP appears to be strain specific; for example, the substrain FSDLZ20M4 exacerbates intestinal inflammation, whereas FSDLZ36M5 alleviates it by regulating immune defenses and protein synthesis 72.

On the other hand, PCs act as major protective agents for the intestinal milieu by producing a diverse array of AMPs, such as α-defensins, lysozyme, and regenerating islet-derived proteins 73. Like those in GCs, which exhibit region specificity, PCs levels and the production of α-defensins are significantly greater in the ileum than in the duodenum 74, indicating that the duodenum exhibits inferior resistance to microorganisms, thus increasing susceptibility to infection in AP patients. During the malignant progression of AP, the differentiation of PCs is commonly suppressed, leading to the disruption of intestinal homeostasis and exacerbation of pancreatic injury. Fu *et al.* revealed that a PCs dysfunction was evident in both AP patients and experimental AP mice, and that lysozyme supplementation partially restored PCs function, reducing the severity of AP and gut microbiota dysbiosis 75. Additionally, the relative abundance of *Escherichia-Shigella* bacteria and the severity of histopathological pancreatic injury are negatively correlated with a decrease in lysozyme levels in AP 76. The administration of dithizone or other drugs to deplete intestinal PCs and exacerbate pancreatic and intestinal injury led to a significant increase in the abundance of pathogenic bacteria (*e.g., Firmicutes*, *Helicobacter pylori*, and* Escherichia-Shigella*) and a decrease in beneficial bacteria (*e.g., Bacteroidetes* and* Blautia*) in the intestinal tract of AP model mice 77. Treatment of AP mice with *L. reuteri* restored intestinal barrier function and ameliorated pancreatic injury by stimulating the differentiation of PCs in a NOD2-dependent manner 16. In addition, the secretion of AMPs by PCs plays a crucial role in the maintenance of gut homeostasis. Huang and colleagues reported that the levels of lysozyme and α-defensins secreted by PCs were significantly decreased, whereas the abundance of *Escherichia-Shigella* was increased in ANP patients, and supplementation with AMPs partially ameliorated pancreatic and intestinal inflammation and injury 78. These findings indicate that maintaining the abundance of PCs in the intestine protects against SAP.

### 3.3 Immune intestinal barrier integrity

**Figure 2:**
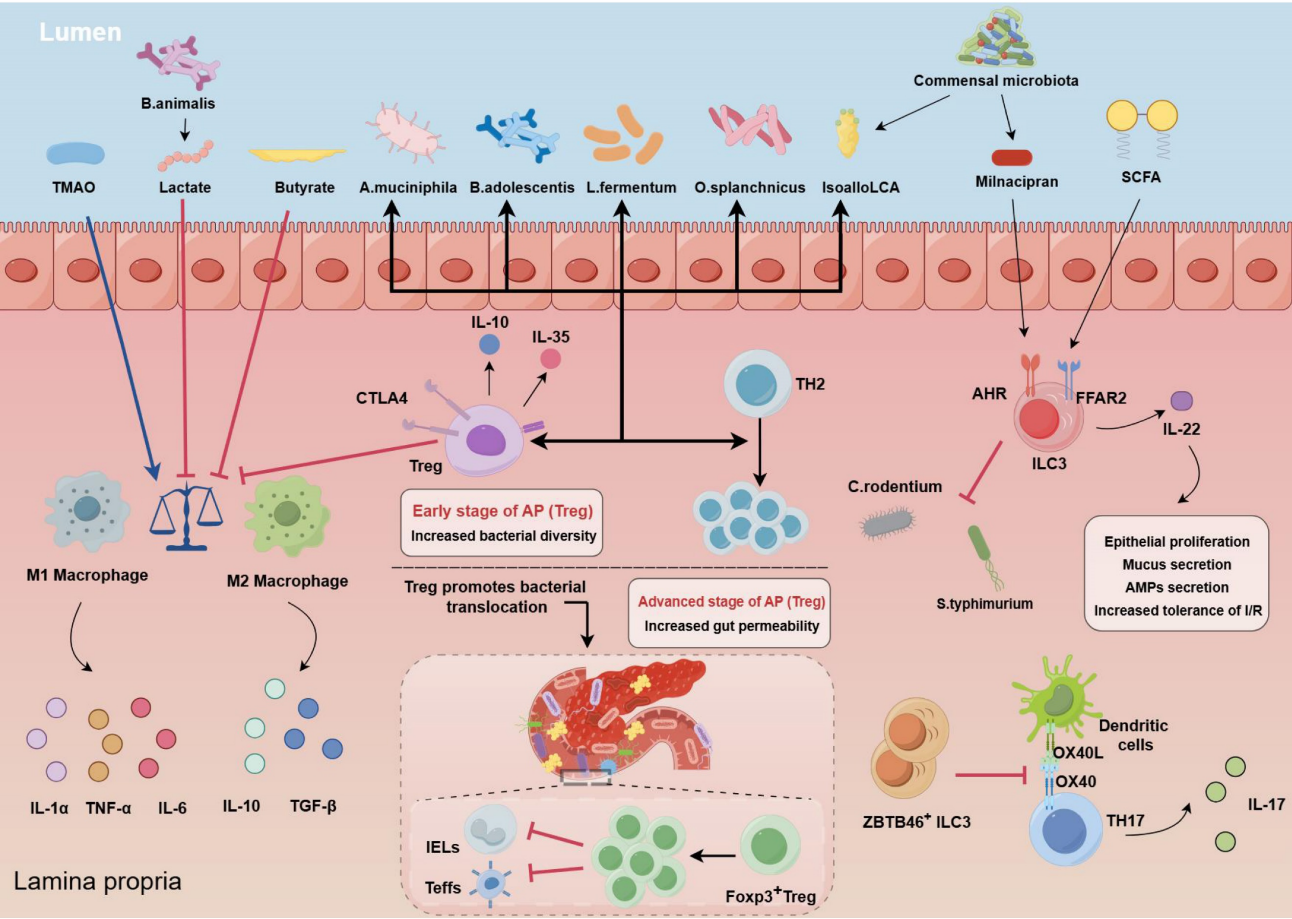
** Immune imbalance in AP progression.** In AP, macrophages often shift towards the M1 phenotype, but this trend can be countered with supplements such as *Bifidobacterium,* lactate, TMAO and butyrate. The FFAR2 on ILC3 recognizes SCFAs, leading to IL-22 secretion which promotes epithelial growth, mucus production, and antimicrobial peptide secretion. Gut microbiota signals facilitate differentiation of specific ILC3 cells, which in turn inhibit the growth of Th17 cells. ILC3 cells also defend against infections like *C. rodentium* and *S. typhimurium* and help maintain the balance between Treg and Th17 cells, and contributing to immune homeostasis. FOXP3^+^RORγt^+^ Treg cells help reduce inflammation by pushing macrophages towards the M2 phenotype, increasing CTLA-4 expression, and releasing anti-inflammatory molecules such as IL-10 and IL-35. Probiotics and bacterial metabolites like isoallo-LCA also boost gut microbiota diversity and increase these Treg cells. However, in advanced AP, excessive Treg cells can overly suppress the immune response, hindering other immune cells in the gut and weakening the intestinal barrier, allowing potential pathogens to infiltrate. (TMAO: trimethylamine-N-oxide; SCFAs: short-chain fatty acids; FFAR2: free fatty acid receptor 2; ILC3: type 3 innate lymphoid cells; AHR: aryl hydrocarbon receptor; CTLA-4: cytotoxic T lymphocyte-associated antigen 4; Treg: Regulatory T cells).

The immune barrier represents the ultimate line of defense in intestinal protection. In the early stage of AP, systemic inflammatory response syndrome (SIRS) is dominant, with excessive inflammatory activation and cascade amplification injuring the pancreas and intestines; subsequently, SIRS enters a remission period, where anti-inflammatory responses gradually take over, leading to compensatory anti-inflammatory response syndrome (CARS), allowing intestinal bacterial translocation to other organs and resulting in secondary infections, thus transforming the intestine from a victim into an accomplice 12, 79, 80. The gut microbiota and its derivatives can act as PAMPs, which are specifically recognized by PRRs, participating in innate and adaptive immunity and amplifying the inflammatory response in AP (Figure 2).

*Intestinal macrophages and neutrophils are essential for regulating immune homeostasis*. In the initial phases of AP, the pancreas is infiltrated by innate immune cells, including neutrophils, macrophages, and ILCs 49. Among these cells, macrophages and neutrophils are pivotal components of the microbiota‒gut‒pancreas axis and are closely associated with excessive inflammatory activation and cascade amplification in AP. *Parabacteroides* can alleviate the inflammatory response in an AP experimental model by producing acetate to limit neutrophil infiltration 10. *Bifidobacterium* and its metabolite lactic acid inhibited the aberrant differentiation of M1 macrophages, which was induced by inflammatory responses, in the pancreas and spleen 20; the metabolite trimethylamine N-oxide promoted macrophage inflammatory polarization by stimulating NLRP3 expression 49. The membrane protein Amuc_1100, which is secreted by *Akkermansia muciniphila*, alleviates the severity of AP through its anti-inflammatory properties, reducing the infiltration of Ly6C^+^ macrophages and neutrophils by modulating the composition of the gut microbiota and tryptophan metabolism 59. The administration of GV-971 (sodium oligomannate) significantly inhibited macrophage M1 polarization and subsequent lethal inflammation by blocking the MAPK pathway while also enhancing both the peripheral and intestinal immune systems through the modulation of the gut microbiota 81. Conversely, an increased abundance of pathogenic bacteria in the gut was positively correlated with pancreatic and systemic immune cell infiltration.

*Dysregulation of the Treg/Th17 balance exacerbates AP*. The Treg/Th17 balance is essential for maintaining intestinal barrier function and tissue homeostasis, and alterations in this balance are intricately linked to the collaboration between immune cells and commensal intestinal bacteria 82, 83. Treg cells influence the differentiation and functionality of immune cells via diverse mechanisms and have potent anti-inflammatory effects. A significant increase in the number of circulating CD4^+^CD25^+^CD127^low^ Tregs was associated with an increased risk of infectious complications and mortality in SAP patients 84. In contrast, patients with SAP who develop MOF tend to have lower levels of circulating CD4^+^CD25^+^CD127^high^ Tregs 85. Immunosuppression, increased Tregs, and related inflammatory cytokines contribute to the complex interactions that lead to inflammation and infection in patients with SAP, primarily due to intestinal mucosal barrier dysfunction 86. During the period of AP exacerbation, there is a gradual increase in Treg cells in the gut, which might be interpreted as an adaptive response to counteract inflammation. In addition, dysbiosis of the gut microbiota can influence the progression of acute pancreatitis by regulating the differentiation of Tregs. It has been demonstrated that parthenolide improves intestinal inflammation by modulating the Treg/Th17 balance in a gut microbiota-dependent manner 87. Additionally, *B. adolescentis* is able to ameliorate chronic colitis by inducing a protective Treg/Th2 response and remodeling the gut microbiota 88. Overactivation of Treg cells led to immunosuppressive responses and inhibited the differentiation of effector T cells and CD8α^+^γδTCR^+^ intraepithelial lymphocytes within the lamina propria, disrupting the intestinal barrier and facilitating the translocation of facultative pathogens, which might have contributed to ANP 12. However, few studies have investigated the interplay between alterations in the intestinal microflora and Treg cells during the progression of AP. We need to further investigate the relationship between the dynamic evolution of regulatory Tregs and changes in intestinal flora, with the objective of potentially regulating the immune imbalance in AP.

## 4. Potential mechanisms underlying the disruption of intestinal homeostasis induced by SAP

### 4.1 The mechanism of bacterial transmission in SAP

More than half of AP patients exhibit intestinal barrier dysfunction, which is significantly associated with poorer clinical outcomes and prolonged hospital stays 89. The intestinal microbiota breaks the compromised gut barrier, exacerbating systemic or local inflammation and leading to secondary infections, resulting in a “second strike” for AP patients. An increased abundance of *Escherichia-Shigella*, *Enterococcus*, or *Staphylococcus* stimulates Treg differentiation, leading to a Treg/Th17 imbalance, while also increasing the levels of proinflammatory intestinal factors that compromise intestinal permeability and result in secondary infections of necrotic pancreatic tissue 4, 12. The precise mechanisms of bacterial metastasis processes in AP remain largely unexplored, but significant impairment of intestinal barrier function is a critical prerequisite for bacterial translocation.

One of the possible mechanisms of bacterial translocation involves bacteria or their endotoxins breaching the intestinal mucosal barrier, infiltrating the mesenteric lymph node and/or portal vein system, and disseminating to injured organs via the systemic circulation 90. High concentrations of pathogenic bacteria, such as *E. coli* and *Enterococcus*, have been detected in the peripheral blood and the drainage fluid of SAP patients, and shifts in the abundances of these bacteria are significantly correlated with the levels of neutrophils and inflammatory factors in patients 4, 25, 91. Furthermore, the retrograde transport of duodenal bacteria to the damaged pancreas via the pancreatic duct may constitute another potential process of bacterial metastasis in AP 70, 92. In SAP, the microbiota in the duodenum is dramatically altered compared with that in the cecum and colon, and an imbalance in intestinal immunity results in *Escherichia-Shigella* translocation from the duodenum to the pancreas 12. In addition, colonization by gut bacteria in the pancreas has also been observed in pancreatic ductal adenocarcinoma, which has been confirmed by colocalization analysis and fluorescence-labeled bacteria 93. Owing to its anatomical structure, the pancreatic duct and bile duct converge and open at the large duodenal papilla, which obviously provides the opportunity for the intestinal flora to retrograde, especially the duodenal bacteria, which have more opportunities to colonize the pancreas through the pancreatic duct.

Although the mechanisms underlying gut microbiota dysbiosis and bacterial translocation require further exploration, there is a consensus that repairing intestinal damage is crucial for improving the progression of AP. Supplementation with biochanin A significantly mitigated AP-associated barrier damage by increasing the expression of TJ proteins, decreasing the translocation of *E. coli* to the pancreas, inhibiting TLR4-MAPK/NF-κB and NLRP3 inflammasome activation, and alleviating AP-associated tissue damage 94. Additionally, increased lysozyme secretion from Paneth cells or mucin-2 secretion from goblet cells can activate the Wnt pathway and the expression of Lgr5 in intestinal stem cells, promoting intestinal homeostasis and contributing to the remission of AP 65, 75. Similarly, the inhibition of phosphoenolpyruvate kinase 1 expression in IECs leads to the upregulation of TJs and the restoration of lysozyme and MUC2 secretion, ultimately alleviating AP 95. Therefore, future studies aimed at alleviating AP should focus on repairing intestinal mucosal damage, adjusting intestinal motility, implementing selective digestive decontamination, inhibiting small intestinal bacterial overgrowth, and utilizing bacterial tracers to further elucidate the role of bacterial translocation in AP.

### 4.2 Gut microorganisms and metabolites intricately modulate multiple signaling pathways during AP progression

**Figure 3:**
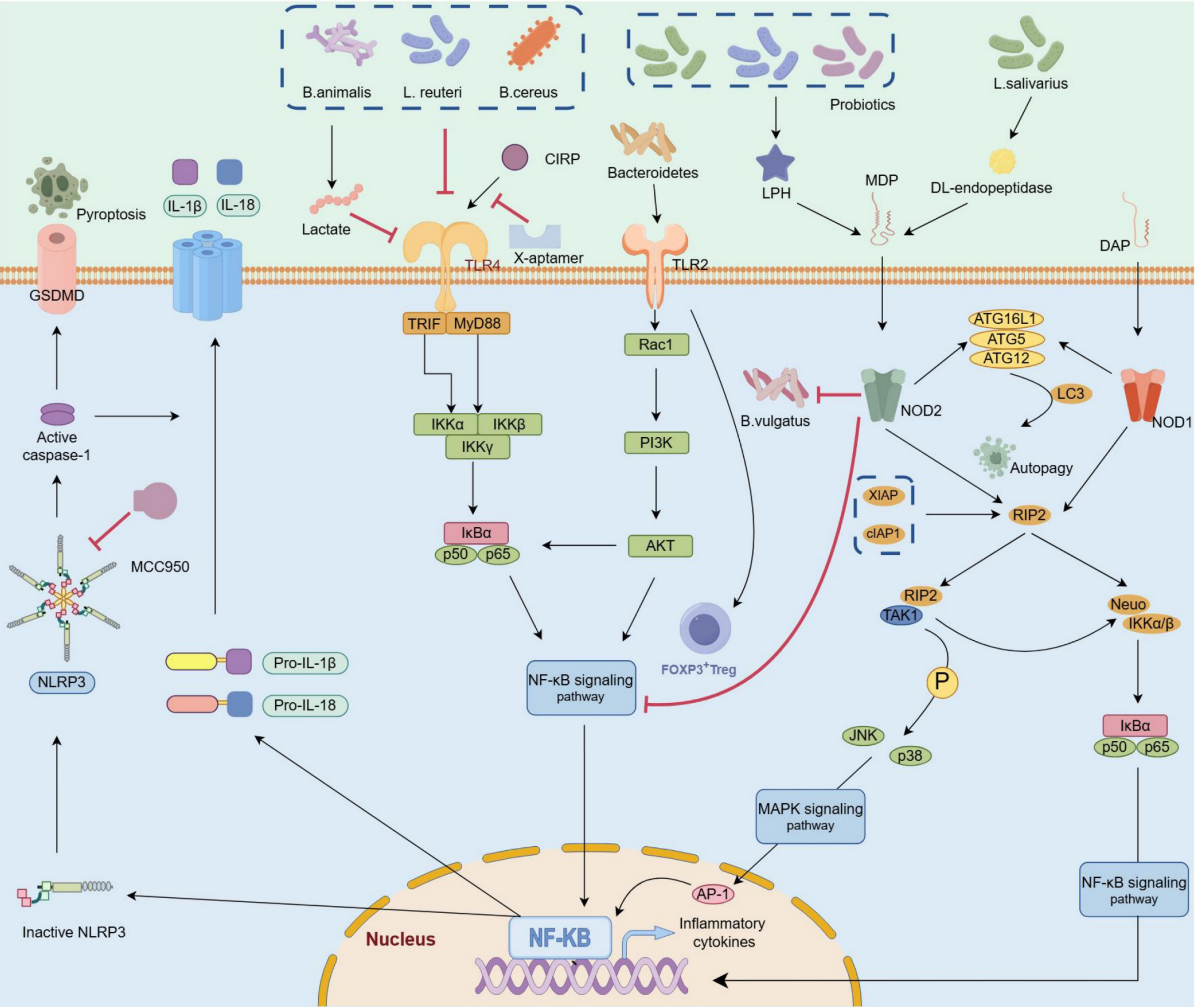
** Gut microbes and metabolites mediate various signaling pathways related to AP progression.** TLRs identify signals from gut bacteria, activating the MyD88/NF-κB signaling pathway, promoting inflammation and autophagy in AP. Supplementing beneficial probiotics such as *B. animalis*, *L. reuteri,* or *B. cereus* can reduce inflammation-induced damage by inhibiting the MyD88/NF-κB signaling pathway. The NOD1 receptor inside cells connects to DAP from gram-negative bacteria, activating the NOD1/RIP2/NF-κB pathway to drive inflammation. NOD2, detects MDP generated by DL-endopeptidase, thereby activating the NF-κB and MAPK signaling pathways and promoting the expression of inflammatory genes. Interestingly, NOD2 activation also counters inflammation by suppressing the TLR-mediated NF-κB and MAPK pathways. Probiotics generating MDP can reduce inflammation, and supplements like L. salivarius or mifamurtide can compensate for lacking DL-endopeptidase. The NLRP3 inflammasome, influenced by these pathways, promotes inflammatory cytokine release and cell death by activating caspase-1. The compound MCC950 and *B. cereus* can suppress NLRP3, inhibiting harmful cell death processes. (TLRs: toll like receptors 2; NOD1: nucleotide binding oligomerization domain containing 1; NOD2: nucleotide binding oligomerization domain containing 2; DAP: diaminopimelic acid; MDP: muramyl dipeptide).

There is bidirectional crosstalk between the pancreas and the intestinal flora. On the one hand, alterations in pancreatic function result in significant shifts in the intestinal microbial composition 96. AMPs produced by the pancreas can modify the gut microbiota profile upon entry into the intestine. The absence of the Ca^2+^ channel Orai1 in pancreatic acinar cells led to a decrease in AMP secretion, consequently fostering the proliferation and dysbiosis of intestinal microbes, which could be reversed by supplementation with cathelicidin-related antimicrobial peptides 97. Research involving mouse models, porcine models and humans has consistently demonstrated that exocrine pancreatic insufficiency precipitates disturbances in the gut microbiota 98. Furthermore, the administration of pancreatic enzymes enriched populations of beneficial bacteria, such as *Akkermansia muciniphila* and *Lactobacillus reuteri*, diminished the presence of pathogenic species such as *Escherichia/Shigella*, *Acinetobacter*, and *Stenotrophomonas*, and influenced the profile of bacterial metabolites detected in the bloodstream 99-101. Contrarily, the gut microbiota and its metabolites regulate numerous signaling pathways during AP progression. Infected or colonized bacteria in humans can be recognized by the pattern recognition receptors (PRRs) of innate immune cells or ILCs through physical contact, toxin production, metabolite synthesis, or protein secretion, referred to as microbe-associated molecular patterns, to mediate signal transduction and immune responses in the host (Figure 3). PRRs primarily consist of extracellular TLRs or intracellular NOD-like receptors (NLRs), which fulfill the host's requirements for bacterial recognition and interaction 102. If PRRs are activated by pathogens, they orchestrate the immune responses of the host against these pathogens, whereas in the presence of intestinal commensal bacteria, PRRs are responsible for maintaining immune tolerance. The classification and recognition of these bacteria strongly influence intestinal defense capabilities.

*TLRs*. TLRs are single transmembrane noncatalytic proteins that are widely expressed in IECs and innate immune cells, among which TLR2, TLR4 and TLR9 are involved in bacterial recognition. TLR4, a pivotal membrane receptor in AP, protects against pancreatic and lung injury by promoting NLRP3 activation and TLR4/MyD88/NF-κB signaling transmission 94, 103. The colonization of *Bifidobacterium animalis* and reinforcement of the biosynthesis of its metabolite lactate acid in the intestinal tract of mice attenuated TLR4/MyD88 signal activation, resulting in reduced release of proinflammatory factors, inhibition of M1 macrophage infiltration in the pancreas, and alleviation of macrophage-associated local and systemic inflammatory responses 20. In contrast, compared with wild-type AP model mice, TLR4ΔIEC model mice presented more pronounced pancreatic injury and systemic inflammatory responses, accompanied by dramatic dysbiosis of the intestinal microbiota, whereas *L. reuteri* administration ameliorated tissue injury and inflammatory responses in TLR4ΔIEC mice 16. These findings indicate that unlike in other tissues, sustaining an inflammatory response in the intestine during the advanced stage of AP may mitigate the risk of complications such as pancreatic necrosis caused by ectopic flora.

Although the involvement of TLR4 in AP deterioration has been widely established, the role of TLR2 in AP remains controversial. For example, a study revealed that the expression of TLR2 in the pancreas and ileum did not significantly change during AP progression 16. Additionally, Awla and colleagues induced AP in wild-type, *Tlr2-*deficient and *Tlr4*-deficient mice and reported that *Tlr4,* but not *Tlr2,* regulated chemokine formation, neutrophil recruitment and tissue damage in a SAP mouse model 104. However, recent studies demonstrated that *Tlr2* deficiency in *Tlr2^-/-^* mice significantly ameliorated pancreatic and pulmonary injury, increased the infiltration of neutrophils and macrophages, and markedly reduced the expression levels of NF-κB and NLRP3 in both MAP and SAP models 105, 106. Microorganisms can specifically activate TLR2 and induce the recruitment of FoxP3^+^ Treg cells to the colonic lamina propria, suggesting that the recognition of the microbiota by TLR2 may serve as a potential mechanism for altering mucosal tolerance 107. Hence, further investigation into the role of TLR2 is imperative, particularly in terms of elucidating the underlying biological mechanisms governing its interaction with the intestinal microbiota across different disease stages.

*NLRs*. TLRs primarily recognize extracellular pathogens and related substances, whereas intracellular bacteria and viruses are predominantly recognized by the cytoplasmic innate immune receptor NLR, which stimulates the NF-κB- and MAPK-related pathways and facilitates proinflammatory cytokine secretion 108. NOD1, the major subtype of NLR, is widely expressed in a variety of cells and tissues in humans. NOD1 is specifically activated by the diaminopimelic acid fragment in gram-negative bacteria and subsequently mediates NOD1/RIP2/NF-kB signaling to promote inflammation. Interestingly, the expression levels of NOD1 are significantly increased in SAP-induced intestinal injury 109, 110, and interventions targeting this pathway significantly ameliorate pancreatic and intestinal injury and reduce the severity of SIRS 111.

Notably, NOD2 plays dual roles in inflammatory diseases. On the one hand, NOD2 specifically recognizes muramyl dipeptide, which activates the NF-kB and MAPK signaling pathways and promotes the expression of inflammatory-related genes and cytokines 112; on the other hand, NOD2 suppresses the TLR-mediated NF-kB and MAPK pathways and exerts anti-inflammatory effects 108. A previous study reported that *Bacteroides vulgatus* could inhibit CD82 activity, promote BRCC3-dependent K63 deubiquitination and increase the activation of the NLRP3 inflammasome, thereby alleviating intestinal injury induced by an overstimulated inflammatory response in the colon 113. However, the interactive mechanisms between the intestinal microbiota and NOD2 in AP are largely unclear and deserve further exploration.

Anti-inflammatory and proinflammatory responses are simultaneously stimulated in AP, and NLRP3 is a key cytosolic immune factor that responds to cellular stress signals and is involved in orchestrating immune responses and establishing intestinal homeostasis. Convincing evidence has demonstrated that NLRP3 is involved in the activation of adaptive immunity in AP. Treatment with MCC950 (an NLRP3 inhibitor) reduces the infiltration of neutrophils and macrophages and dampens pancreatic and systemic inflammatory responses 79, 105. Additionally, prophylactic treatment with antibiotics prevents the amplification of AP via the intestinal‒pancreatic axis by restraining the activation of TLR4/NLRP3 signaling in the colon, downregulating the expression of proinflammatory factors, promoting the expression of intestinal TJ-related proteins, improving the morphology of the intestine, and reducing the translocation of intestinal bacteria 114. Previous studies have demonstrated that *Bifidobacterium animalis*^20^ and *Bacillus cereus*^115^ exert anti-inflammatory effects by suppressing TLR4/NF-κB/NLRP3 signaling, resulting in a reduction in intestinal barrier damage, weakened local and systemic inflammatory responses and decreased incidence of pancreatic edema, hemorrhage, and necrosis complications. Despite the proinflammatory effects of NLRP3 in diseases, it also contributes to the activation and functions of anti-inflammatory immune cells. Sendler *et al.* reported a significant decrease in Treg cells and an attenuated Th2-mediated immune response in NLRP3-deficient AP model mice 79.

Moreover, alterations in the interactions among the gut microbiota, the metabolic community, and the expression of crucial genes in the host are key in the cooperative regulation of AP progression. During the recovery stage of AP, NLRP3 activity decreases in antibiotic-treated and germ-free AP mice, whereas reactivation of NLRP3 occurs in mice receiving FMT from AP patients, leading to exacerbated systemic inflammation 25. Despite numerous studies demonstrating the role of NLRP6 in various inflammatory disease processes, few studies have elucidated its role in AP.

## 5. Exploring novel strategies for improving the prognosis of patients with AP by regulating the gut microbiota

### 5.1 Probiotics

Currently, numerous experimental and clinical studies have indicated that changes in the gut microbiome can have a broad range of effects throughout the body. Hence, as shown in Table 3, probiotics have been employed as adjunctive therapies for various diseases; probiotics modulate the gut microbiota and alleviate symptoms 116. In an AP mouse model, supplementation with *Bifidobacterium animalis* ameliorated macrophage-related inflammatory responses and attenuated acinar cell necrosis 20; furthermore, *Lactobacillus* can modulate innate immune homeostasis, preserve PCs functionality, promote AMP secretion through increased tryptophan metabolism, and ameliorate pancreatic and intestinal edema and necrosis 117, 118. The commensal flora *Bifidobacterium* is widely used to evaluate its therapeutic effects on inflammatory diseases in experimental and clinical trials. Researchers have administered artificial-enzyme-armed *Bifidobacterium longum* to mice, and the treatment increased the amount of reactive oxygen species scavenged, attenuated the level of inflammatory mediators, and modulated the intestinal microbiota 119. Additionally, an RCT was performed to assess the effectiveness of *Bifidobacterium* quadruple living bacterium in 60 SAP patients; the incidence of MOF was significantly reduced and the duration of abdominal pain and hospital stay were significantly shortened, but the death rate was not significantly different 120. However, in a multicenter randomized, double-blind, placebo-controlled trial with 298 predicted SAP patients, patients who were administered a multispecies probiotic preparation presented higher rates of infectious complications, deaths, and bowel ischemia than did those in the placebo group 121. A meta-analysis indicated that the treatment of SAP patients with probiotics slightly influences infection or mortality rates during advanced disease stages 122. We believe that these inconsistent outcomes might be the result of each study applying a unique starting time, dose, mode, duration of probiotic administration, and specific degree of clinical adjuvant therapy. Therefore, subsequent studies should define the use of probiotics more strictly.

### 5.2 Prebiotics

Prebiotics are indigestible carbohydrate substrates that improve intestinal function by promoting the proliferation of beneficial bacteria, modulating adaptive immunity, and enhancing mineral absorption. As shown in Table 4, lactulose has garnered significant attention because it can increase the abundance of beneficial bacteria such as *Bifidobacterium*, *Lactobacillus*, and *Muribaculum*, to regulate intestinal immune homeostasis 123, 124. In addition, lactose is metabolized into lactic acid in the intestine in a dose-dependent manner to decrease serum amylase levels, promote the proliferation of intestinal stem cells and PCs and alleviate intestinal mucosal injury 125, 126. Interestingly, in a randomized controlled trial (RCT) involving MSAP patients complicated with gut dysfunction, participants treated with lactose had lower serum cytokine levels and gut permeability indices than patients treated with the Chinese herb rhubarb 48.

### 5.3 Synbiotics

Synbiotics, which consist of a combination of probiotics and prebiotics, are formulated to increase their efficacy and durability. The combination of *Bifidobacterium* and lactulose increases the metabolism of tryptophan in the intestine to help maintain gut homeostasis 127, and the administration of *Bacillus coagulans* and lactulose significantly ameliorated intestinal barrier permeability damage and inhibited apoptotic cell death 128. However, a prospective and double-blind RCT indicated that the administration of synbiotics to SAP patients did not significantly reduce complications, mortality, or intervention rates; however, the length of hospital stay was reduced 129.

### 5.4 FMT

A broad range of clinical and animal experiments indicate that FMT is a potential therapeutic strategy for limiting inflammation and organ damage in various diseases FMT is achieved by implanting fecal material from healthy donors into the patient's intestine to alleviate gut microbiota dysbiosis 130-132. However, the therapeutic efficacy of FMT remains controversial due to the heterogeneity of the fecal microbiota, variations in receptor reactivity across different samples, and unique inflammatory characteristics at different stages of AP 133. Liu *et al.* reported that FMT in AP mice ameliorated the dysbiosis of gut microbiota communities, decreased mitochondrial dysfunction and the oxidative stress response, and reshaped the intestinal ecological balance 134. Li *et al.* demonstrated that pancreatic injury and systemic inflammation were alleviated and that the intestinal NLRP3 inflammasome was stimulated in antibiotic-treated and germ-free mice. Nevertheless, FMT can reactivate the NLRP3 inflammasome and exacerbate AP 25. After FMT in ANP mice, the mortality rate nearly doubled, accompanied by a significant increase in the number of pancreatic colony-forming units 15. Thus, FMT is not currently recommended for the treatment of AP in clinical practice, and further studies are needed to assess the effect of FMT and decipher the underlying mechanisms of FMT as a treatment for AP.

## 6. Future perspectives

Significant advancements have been made in the investigation of the interactions between the gut microbiota and the pancreas via the “microbiota‒gut‒pancreas” axis. The intestinal flora and pancreatic tissue-resident microbes present unique spectrum in AP patients with different etiologies and severities, and novel therapeutic approaches targeting the gut microbiota generate differing outcomes owing to variations in clinical implementation protocols. Well-designed clinical trials are urgently needed to evaluate the dosage and formation of the multispecies probiotic preparation and the duration of treatment for AP.

## Figures and Tables

**Table 1:** Representative animal studies demonstrating the effectiveness and mechanism of candidate gut bacteria in AP progression

Tested sample	AP-related Bacteria	Rodent strains	Methods of AP model establishment	Sequencing methods	Main finding	Ref	
Fecal	(↑): *Escherichia-Shigella, Enterococcus, Proteobacteria* (↓): *Prevotella, Bacteroides, Bifidobacterium, Lachnospiraceae*	Male C57BL/6 mouse (n = 40)	Intraperitoneally, 100 μg/kg body weight, 10 times at 1h intervals, administrate with LPS (10 mg/kg) after final injection of caerulein.	16S rRNA sequencing	The disturbed microbiota was closely correlated with AP severity; the antibiotic-treated mice and germ-free mice exhibited alleviated pancreatic injury after AP induction and FMT in turn exacerbated the disease.	4	
Fecal	(↑): *Escherichia-Shigella, Enterococcus, Firmicutes* (↓): *Muribaculaceae, Bacteroides*	Male C57BL/6 mouse (n = 28)	Intraperitoneally, 100 μg/kg body weight, 10 times at 1h intervals, and administrate with LPS (5 mg/kg) after final injection of caerulein.		Administration with chitosan oligosaccharides alleviated SAP by suppressed the oxidative stress and restoring intestinal homeostasis in the pancreas and ileums.	26	
Fecal	(↑): *Firmicutes* (↓): *Bacteroides, Parabacteroides*	C57BL/6 mouse (n = 10) Hpa-Tg mouse (n = 10)	Intraperitoneally, 50 μg/kg body weight, 8 times at 1h intervals.		Parabacteroides produced acetate to alleviate AP by reducing neutrophil infiltration.	10	
Fecal	(↓):* Helicobacter, Lachnospiraceae*	C57BL/6 mouse (n = 42) rIL-22 mice (n = 12)	5% Taurocholic acid induced AP model: 10 μl/mice, 5μl/min, and the bile duct was occluded by noninvasive microslip.		Early stage decreased colonic IL-22 aggravates intestinal mucosal barrier dysfunction and microbiota dysbiosis in SAP.	22	
Fecal	(↓): *Muribaculacea, Lactobacillus*	Male C57BL/6 mouse (n = 42) TLR4ΔIEC mouse (n = 12).	8% L-arginine induced AP model: intraperitoneally, 4.0 g/kg body weight, 2 times at 1h intervals.		Loss of intestinal epithelial TLR4 exacerbated pancreatic and intestinal damage during AP via gut microbiota dysbiosis; *L. reuteri* maintained intestinal homeostasis via Paneth cells modulation.	16	
Fecal	(↑): *Escherichia-Shigella, Enterococcus* (↓):* Lachnospiraceae*	C57BL/6 mouse (n = 34) DEREG moues (n = 16)	Intraperitoneally, 50 μg/kg body weight, 1 time, and partial pancreatic duct ligation.		Treg-activation disturbed the duodenal barrier function and permited translocation of commensal bacteria into pancreatic necrosis.	12	
Duodenal mucosa	(↓): *Prevotella, Actinobacillus*	C57BL/6 mouse (n = 150)	1) MAP: intraperitoneally, Caerulein, 25 μg/kg body weight, 10 times at 1h intervals; 2) SAP: 8% L-arginine, 4.5 g/kg body weight, 2 times at 2h intervals.		MAP mild duodenal barrier dysfunction and slight change in duodenal mucosal microbiota.	17	
Fecal	(↑):* Escherichia-Shigella, Enterococcu* (↓): *Prevotella, Akkermansia, Bacteroide, Parabacteroides*	C57BL/6 mouse (n = 28)	Intraperitoneally, 100 μg/kg body weight, 10 times at 1h intervals, and administrate with LPS (10 mg/kg) after final injection of caerulein.		AMPK/NF-κB/NLRP3 pathway mediated by SCFAs along the gut-lung axis play an essential role in preventing the pathogenesis of SAP.	14	
Fecal	(↑): *Firmicutes* (↓): *Bacteroides, Actinobacillus*	Male C57BL/6 mouse (n = 12)	Intraperitoneally, 100 μg/kg body weight, 7 times at 1h intervals, and administrate with LPS (10 mg/kg) after final injection of caerulein.	Metagenomics	Identified the relationship between the gut microbiome and metabolite levels during AP.	45	

Fecal	(↑): *Escherichia-Shigella* (↓):* Lactobacillus, Lachnospiraceae*	C57BL/6 mouse (n = 20) Rftn1 deficient mouse (n = 20)	5% Taurocholic acid induced AP model: 0.1ml/100g body weight, retrograde injection.	16S rDNA sequencing	Inhibiting M1 macrophages activation, reduce pancreatic lesions and reduce intestinal damage	19	
Fecal	(↑):* Enterococcus* (↓):* Akkermansia, Bifidobacterium*	C57BL/6 mouse (n = 42) TLR4 deficient mouse (n = 12) Nlrp3 deficient mouse (n = 12) Myd88 deficient mouse (n = 12)	1) MAP: intraperitoneally, 25 μg/kg body weight, 7 times at 1h intervals; 2) SAP: intraperitoneally, 25 μg/kg body weight, 10 times at 1h intervals, and administrate with LPS (7.5 mg/kg) after final injection of caerulein.		*B. animalis* relieved macrophage-associated local and systemic inflammation of AP in a TLR4/MyD88- and NLRP3/Caspase1-dependent manner through its metabolite lactate.	20	
Fecal	(↑): *Helicobacter* (↓):* Muribaculaceae*	Sprague-Dawley rat (n = 64)	3.5% Taurocholic acid induced AP model: 0.1ml/100g body weight, retrograde injection.		Qingyi granules modulated the gut microbiota and metabolic abnormalities to ameliorate SAP.	5	

Notes: 1) Hpa-Tg mice: Heparanase transgenic mice; 2) rIL-22 mice: Administration of recombinant IL-22 mice; 3) TLR4ΔIEC mice: Intestinal epithelial TLR4 knockout mice; 4) MAP: mild acute pancreatitis; 5) SAP: severe acute pancreatitis.

**Table 2:** Representative clinical human studies demonstrating the effectiveness and mechanism of candidate gut bacteria in AP progression

Tested sample	AP-related bacteria	Subjects	Sequencing methods	Main finding	Ref
Duodenal mucosa	(↑):* Streptococcus* (↓): *Prevotella*	MAP patients (n = 16), Healthy controls (n = 16).	16S rRNA sequencing	MAP patients with mild duodenal barrier dysfunction and slight changes in duodenal mucosal microbiota.	17
Fecal	(↑): *Escherichia-Shigella, Enterococcus, Proteobacteria* (↓): *Blautia, Prevotella, Bacteroides, Faecalibacterium*	MAP patients (n = 41), MSAP patients (n = 59), SAP patients (n = 30), Healthy controls (n = 35).		The disturbed microbiota was closely correlated with AP severity; the antibiotic-treated mice and germ-free mice exhibited alleviated pancreatic injury after AP induction and FMT in turn exacerbated the disease.	4
Fecal	(↑): *Escherichia-Shigella, Enterococcus* (↓): *Blautia, Bifidobacterium*	ANP patients (n = 19), non-ANP patients (n = 39), Healthy control (n = 20).		Healthy, ANP and non-ANP patients presented distinct gut microorganism composition	21
Fecal	(↑): *Escherichia-Shigella, Enterococcus, Proteobacteria, Blautia, Bifidobacterium, Ruminococcus*	AP patients (n = 65), Healthy controls (n = 20).		Healthy, AP-ARDS and AP-nonARDS patients presented distinct gut microorganism composition and functions.	37
Fecal	(↑): *Enterococcus* (↓): *Blautia, Ruminococcus, Faecalibacterium*	MAP patients (n = 43), MSAP patients (n = 9), SAP patients (n = 2), Healthy controls (n = 46).	16S rDNA sequencing	AP patients with lower gut microbiome diversity and a higher abundance of sulfidogenic bacteria.	18

Note: 1) AP: Acute pancreatitis; 2) MAP: Mild acute pancreatitis; 3) SAP: Severe acute pancreatitis;4) HTGP: Hypertriglyceridemia-induced acute pancreatitis; 5) AP-ARDS: Acute pancreatitis patients with acute respiratory distress syndrome; 6) NETs: neutrophil extracellular traps.

**Table 3:** Representative studies demonstrating therapeutic effects of probiotics in AP

Probiotics	Subjects or Rodent strains	Treatments	Main findings	Ref
*B.animalis*	C57BL/6 mouse, TLR4 deficient mouse, Nlrp3 deficient mouse, Myd88 deficient mouse (n = 92)	Oral gavage, 1 × 1010 CFUs	*B. animalis* relieved macrophage-associated local and systemic inflammation of AP in a TLR4/MyD88 and NLRP3/Caspase1 dependent manner through its metabolite lactate.	20
*plantarum B7/* *L. rhamnosus L34 and L. paracasei B13*	Male ICR mouse (n = 24)	Oral gavage 6 days, 1 × 108 CFUs	Reducing inflammation and restoring the maintenance of intestinal integrity	117
*L. plantarum* FLPL05	C57BL/6 mouse (n = 20)	Oral gavage, 1 × 107 CFUs	Partially reversing pancreatitis	135
*Lactobacillus* mixture	Male SPF Sprague-Dawley rats (n = 24)	Oral gavage, 4.5 × 109 CFUs	Exhibiting potent antioxidant and anti-inflammatory effects	136
*Lactobacillus acidophilus, Lactobacillus casei, Lactobacillus salivarius, Lactococcus lactis, Bifidobacterium bifidum, Bifidobacterium lactis*	Predicted SAP patients (n = 152)	Enteral administration twice a day for 28 days, 1×10^10^ CFUs	Infectious complications, number of deaths, the risk of bowel ischemia occurred higher; Increasing the risk of infectious complications and risk of mortality	121
*B. bifidum*, *B. acidophilus*, *E. faecalis*, *B. cereus*	SAP patients (n = 120)	Enteral nutrition	Reducing the incidence of multiple organ dysfunction syndrome; shortening the duration of abdominal pain and hospital stay; the death rate was not significantly changed	120

Note: a) ICR: Institute of Cancer Research; b) CFU: Colony-Forming Units; c) SPF: Specific pathogen-free.

**Table 4:** Representative studies demonstrating therapeutic effects of prebiotics in AP

Category	Prebiotics/metabolites	Subjects or Rodent strains	Treatments	Main findings	Ref
SCFAs	Butyrate	C57BL/6 mouse and GPR109A deficient mouse (n = 40)	Oral gavage 7 days, 200mg/kg	Ameliorating pancreatic inflammation; suppression of NLRP3 inflammasome activation and modulation of immune cell infiltration	137
	Butyrate	Male C57BL/6 mouse (n = 32)	Oral gavage7 days, 200mg/kg or 500mg/kg	Inhibiting pancreatic inflammation by maintaining the intestinal barrier and regulating gut microbiota	69
	Butyric acid	AP patient (n = 35); C57BL/6 mouse	Drinking 4 weeks, 100mM	Ameliorating progression towards necrotizing pancreatitis	15
	Butyric acid	C57BL/6 mouse (n = 32)	Drinking 4 weeks, 100mM	Mitigating acute pancreatitis through amelioration of intestinal barrier dysfunction	47
Oligosaccharides	Chitosan oligosaccharide	C57BL/6 mouse (n = 28)	Oral gavage, 200 mg/kg	Reducing oxidative stress and restoring intestinal homeostasis	26
	Lactulose	MSAP patients (n = 73)	Administration of oral solution twice daily; 50 mL,10 mL	Restoring intestinal function, regulating gut microbiota and promoting the production of SCFAs	48
Tryptophan metabolites	Norharman	C57BL/6 mouse (n = 20) Rftn1 deficient mouse (n = 20)	Oral gavage 3 times for 1 day, 100 mg/kg	Inhibiting M1 macrophages activation, reduce pancreatic lesions and reduce intestinal damage	19

## Abbreviations

AP: acute pancreatitis
MAP: mild AP
SAP: severe AP
FMT: fecal microbiota transplantation
PAMPs: pathogen-associated molecular patterns
MOF: multiple organ failure
HTGP: hypertriglyceridemic pancreatitis
ANP: acute necrotizing pancreatitis
SCFAs: short chain fatty acids
IECs: intestinal epithelial cells
GCs: goblet cells
AhR: aryl hydrocarbon receptor
Rftn1: Raftlin 1
FXR: Farnesoid X receptor
TGR5: Takeda G protein-coupled receptor 5
TLR: Toll-like receptor
ECs: enteroendocrine cells
PCs: paneth cells
TJs: tight junctions
MLCK1: long myosin light chain kinase splice variant 1
MUC2: Mucin 2
FCGBP: IgGFc-binding protein
CLCAl: calcium-activated chloride channel regulator 1
GAPs: antigen passages
IgA: immunoglobulin A
Tregs: regulatory T cells
AMPs: antimicrobial peptides
SIRS: systemic inflammatory response syndrome
CARS: compensatory anti-inflammatory response syndrome
ILCs: innate lymphoid cells
RCT: randomized controlled trial
FMN: formononetin
ZO-1: ZO1 tight junction protein
